# Prognostic value of three rapid scoring scales and combined predictors for the assessment of patients with coronavirus disease 2019

**DOI:** 10.1002/nop2.934

**Published:** 2021-06-03

**Authors:** Hai Hu, Weili Kong, Ni Yao, Yanru Qiu, Rong Yao

**Affiliations:** ^1^ International Emergency Medical Team (Sichuan) Emergency Office of West China Hospital Sichuan University Chengdu Sichuan People’s Republic of China; ^2^ Department of Otolaryngology, Head and Neck Surgery West China Hospital Sichuan University Chengdu Sichuan People’s Republic of China; ^3^ Department of Critical Care Medicine COVID‐19 Medical Team (Hubei) of West China Hospital West China Hospital Sichuan University Chengdu People’s Republic of China; ^4^ Oncology Department of Renmin Hospital of Wuhan University (East Campus) COVID‐19 Ward of Renmin Hospital of Wuhan University Wuhan Hubei People’s Republic of China

**Keywords:** COVID‐19, prediction, prognosis, Rapid Scoring Scales, risk factors

## Abstract

**Aim:**

To explore the factors affecting mortality in patients with COVID‐19 and to verify the predictive value of the three rapid scoring scales MEWS, RAPS and REMS.

**Design:**

Cross‐sectional observational study.

**Methods:**

Kaplan–Meier and Cox survival analyses were performed to identify the risk factors associated with COVID‐19‐related death. A ROC curve analysis was used to evaluate the abilities of the three scoring scales to predict the prognosis of COVID‐19 patients.

**Results:**

Age, low blood oxygen saturation level and decreased lymphocyte count were the high risk factors for COVID‐19‐related mortality. The analysis of the abilities of the three scales to predict the prognosis of COVID‐19 patients: The AUC of 0.641 for the RAPS (*p* = .065). The MEWS (AUC = 0.705, *p* = .007), compared with RAPS, the NRI was 0.371(*p* = .03), and the IDI = 0.092 (*p* = .046); The REMS (AUC = 0.841, *p* < .001), compared with MEWS, the NRI was 0.227(*p* = .12), and the IDI=0.09(*p* = .047); The Combining Predictor (AUC = 0.878, *p* < .001), compared with REMS, the NRI was 0.25(*p* = .113), and the IDI=0.02(*p* = .598).

**Conclusion:**

Patients with an old age, low blood oxygen saturation level and decreased lymphocyte count were at a high risk of COVID‐19‐related mortality. Moreover, our analysis revealed that the REMS had a better prognostic ability than the MEWS and RAPS when applied to COVID‐19 patients. Our findings suggest that the REMS can be used as a rapid scoring tool for the early assessment of COVID‐19 severity.

## 1 INTRODUCTION

Coronavirus disease 2019 (COVID‐19) is caused by infection with the severe acute respiratory syndrome coronavirus‐2 (SARS‐CoV‐2) (Cucinotta & Vanelli, 2020). As of 2 September 2020, more than 30,675,675 cases of COVID‐19 and more than 521,622 related deaths have been reported in more than 200 countries and territories worldwide(Cascella et al., 2020) (Worldometer, 2020). In China, various hospitals have established specialized emergency departments as the first line of defence for COVID‐19 patients. However, the wait times in these facilities are prolonged because of the large numbers of patients and relatively insufficient medical resources, and this situation increases the risk of nosocomial infection. Therefore, trends in the spread of COVID‐19 and the outcomes of patients are related to the ability of a specialized emergency department to classify patients accurately (Zhang et al., 2020). Severe COVID‐19 is associated with a high mortality rate, whereas relatively milder cases tend to resolve without intensive intervention. Therefore, the efficiency of COVID‐19 patient classification could best be improved by applying appropriate scoring scales. These scales were designed as screening tools with the aim of reducing the time required to evaluate patients, and their use can greatly improve the quality of care and therapeutic effects and can thus reduce morbidity and mortality (Nogueira et al., 2014).

Various accurate and effective clinical scoring scales have been developed in recent years. For example, the Rapid Acute Physiology Score (RAPS; Table S1) is an abbreviated version of the Acute Physiology and Chronic Health Evaluation II (APACHE II) scoring table, and several studies have found that this model can be used to evaluate patients’ prognosis(Rhee et al., 1987). The Rapid Emergency Medicine Score (REMS), another commonly used clinical scale, comprises the variables contained in RAPS, as well as the patient's arterial oxygen saturation level and age (Table S2). The effectiveness of the REMS model for the assessment of trauma patients has been confirmed in some studies (Olsson, 2003) (Olsson, 2004). The Modified Early Warning Score (MEWS) is also commonly applied to emergency patients (Table S3) and can be used to detect potential disease‐related changes in patients with severe disease at an early stage and enable measures to prevent deterioration (Gardner‐Thorpe et al., 2006). However, no scoring scale has been designated specifically for the prognostic evaluation of COVID‐19 patients. Therefore, this study aimed to assess and compare the prognostic values of the MEWS, RAPS and REMS for the prognostic evaluation of COVID‐19 patients in a specialized emergency department.

## 2 MATERIALS AND METHODS

### 2.1 Study design

This was a retrospective analysis of data obtained from a database of patients admitted by the West China Hospital Medical Team during the anti‐COVID‐19 epidemic period in Wuhan. Initially, a survival analysis was applied to identify the patient‐related risk factors for mortality. Next, the RAPS, REMS and MEWS scoring scales were applied to the data, and their abilities to predict the prognosis of patients were assessed.

The West China Hospital Institutional Review Committee approved the study and waived the requirement for informed consent from the study subjects due to the study design. The study complied with an international ethical guideline for human research, such as the Declaration of Helsinki. The accessed data were anonymized.

### 2.2 Settings and subjects

This study included all adult patients diagnosed with COVID‐19 between February 7 and March 7, 2020 (*N* = 79). For all patients, the following data were retrieved from the database: basic information (sex, age, final diagnosis, and chronic diseases), vital signs (body temperature, heart rate, systolic and diastolic blood pressure, respiratory frequency), consciousness, oxygen saturation level and RAPS/REMS/MEWS scale scores, etc. The outcome variable was the patient's death or survival at discharge. The observation point for survival calculations was set as the discharge time of the last admitted patient.

### 2.3 Statistical analysis

The data analysis was conducted using the IBM Statistical Program for Social Sciences Statistics 20.0 (SPSS; IBM Corp., Armonk, NY, USA) and MedCalc Statistical Software (Version 18.2.1; MedCalc Software Ltd., Ostend, Belgium). Continuous variables are presented as means ±standard deviations, and categorical variables are described as composition ratios (%). The Mann–Whitney *U* test and Fisher's exact test were used to compare the continuous and categorical variables, respectively. Compared with the population mortality in COVID‐19, the mortality rate of this group is 25.3%, and its statistical power was 100%.

The Kaplan–Meier (K–M) and Cox regression methods were used to perform survival analyses and univariate and multivariate survival analyses, respectively, to establish the relationships between potential predictive factors and mortality. After introducing three emergency rapid scoring scales, we calculated the score of each scales, a binary logistic regression analysis was performed to establish a prediction model between the scores and the mortality rate, and the Hosmer–Lemeshow test was used to determine the goodness of fit of the model.

Receiver operating characteristic (ROC), Net Reclassification Improvement (NRI) and Integrated Discrimination Improvement (IDI) were conducted to evaluate the abilities of the RAPS, REMS and MEWS to predict mortality. The areas under the ROC curves (AUCs) were compared using the *Z* test (Hanley–McNeil method): $Z_{\mathrm{AUC}}=\frac{AUC_{1}‐AUC_{2}}{\sqrt{SE_{1}^{2}+SE_{2}^{2}}}$. RAPS, REMS and MEWS were further combined to calculate new prediction data, and the calculation formula was:

β_1_+β_2_*MEWS/RAPS+β_3_*RAPS/REMS. The best demarcation point of each scoring scale was determined as the maximum Youden's index value. Finally, the corresponding accuracy, sensitivity, specificity, positive predictive value and negative predictive value corresponding to the best cut‐off point of each score were calculated. Net Reclassification Improvement (NRI) and Integrated Discrimination Improvement (IDI) are further used to compare the predictive ability of two models. NRI = (New_sensitivity_ + New_specificity_) − (Old_sensitivity_ + Old_specificity_), Z test was conducted to compare NRI value of the models: $Z_{\mathrm{NRI}}=\frac{\mathrm{NRI}}{\sqrt{\frac{B_{1}+C_{1}}{N_{1}^{2}}+\frac{B_{2}+C_{2}}{N_{2}^{2}}}}$. IDI = (P_new,events_ – P_old,events_) − (P_new,non‐events_ – P_old,non‐events_), *Z* test was conducted to compare IDI value of the models: $Z_{\mathrm{IDI}}=\frac{\mathrm{IDI}}{\sqrt{{\left(SE_{\mathrm{events}}\right)}^{2}+{\left(SE_{non‐events}\right)}^{2}}}$.

For all analyses, a *p *< 0.05 was considered to indicate statistical significance.

## 3 RESULTS

Patients in the surviving and deceased groups had mean ages of 56.52 ± 16.97 and 75.05 ± 12.94 years, respectively, and this difference was significant (*p* <.05). Moreover, the two groups differed significantly with respect to sex, systolic blood pressure, oxygen saturation level, white blood cell and lymphocyte counts, and the MEWS, RAPS and REMS scores (all *p* <.05). In contrast, the two groups did not differ significantly in terms of the heart rate, diastolic blood pressure and body temperature (*p* >.05; Table 1).

**TABLE 1:** Difference between alive group and death group

Variable	Alive(*n* = 59)	Death(*n* = 20)	*p*‐value
Female	35 (87.5%)	5 (12.5%)	**0.015** *
Male	24 (61.54%)	15 (38.46%)	
Age	56.52 ± 16.97	75.05 ± 12.94	**<0.001** *
Heart rate	83.60 ± 15.01	87.47 ± 13.56	0.248
Breathe	19.83 ± 2.068	23.21 ± 5.381	**0.006** *
Systolic pressure	130.40 ± 19.68	143.30 ± 24.85	**0.016** *
Diastolic pressure	80.40 ± 10.75	83.95 ± 14.12	0.347
Temperature	36.59 ± 0.45	36.71 ± 0.72	0.388
oxygen saturation	96.40 ± 2.57	86.53 ± 12.46	**<0.001** *
While cell count	5.54 ± 1.91	11.57 ± 9.79	**0.001**
Lymphocyte count	27.56 ± 11.8	8.86 ± 9.48	**<0.001** *
MEWS	1.48 ± 0.87	2.37 ± 1.53	**0.001** *
RAPS	0.80 ± 1.31	1.58 ± 1.71	**0.03** *
REMS	3.90 ± 2.98	7.90 ± 2.81	**<0.001** *
Combined score	6.18 ± 4.47	11.85 ± 5.13	**<0.001** *

Note

Abbreviations: Combined Score: the combined score of MEWS, RAPs, and REMS; MEWS, Modified Early Warning Score; RAPS, Rapid Acute Physiology Score; REMS, Rapid Emergency Medicine Score.

* *p*‐values were calculated by using Wilcoxon rank sum test for continuous variables, and Fisher's exact test for categorical variables.

**FIGURE 1:**
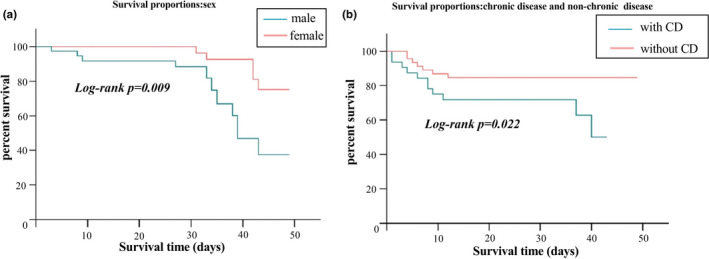
Kaplan–Meier survival estimates according to a. sex and b. chronic disease status

Next, a K–M survival analysis and a univariate Cox regression analysis were performed to identify the variables that differed significantly between the surviving and deceased groups (Table 2). Notably, the patient's age, sex, respiratory frequency, oxygen saturation level, lymphocyte count and chronic disease status were identified as significantly different between the groups (*p* <.05). We chose significant variables after UVA analysis into a subsequent multivariate Cox regression analysis. The patient's age, oxygen saturation level and lymphocyte count were identified as independent risk factors for mortality (*p* <.05). The patients’ survival outcomes classified by sex and chronic disease status are presented in Figure 1a and b, respectively.

**TABLE 2:** Survival‐related factors based on the survival outcomes

Variables	UVA		MVA	
	Adjusted HR (95% CI)	*p*‐value	Adjusted HR (95% CI)	*p*‐value
Age	1.081 (1.09–1.124)	**<0.001**	1.043 (0.99–1.093)	**0.05**
Sex				
F	Reference		Reference	
M	0.278 (0.01–0.78)	**0.014**	1.18 (0.33–4.20)	0.8
Respiration frequency	1.236 (1.125–1.359)	**<0.001**	1.17 (1.02–1.34)	**0.02**
Oxygen saturation level	0.804 (0.794–0.864)	**<0.001**	0.879 (0.802–0.964)	**0.003**
Lymphocyte count	0.89 (0.847–0.936)	**<0.001**	0.94 (0.885–0.999)	**0.04**
Chronic disease				
No	Reference		Reference	
Yes	2.94 (1.15–7.53)	**0.022**	0.341 (0.103–1.125)	0.07

Note

CIs are presented as lower limit–upper limit ranges. Abbreviations: UVA, univariate analysis; MVA, multivariable analysis; HR, hazard ratio; 95% CI, 95% confidence interval.

**FIGURE 2:**
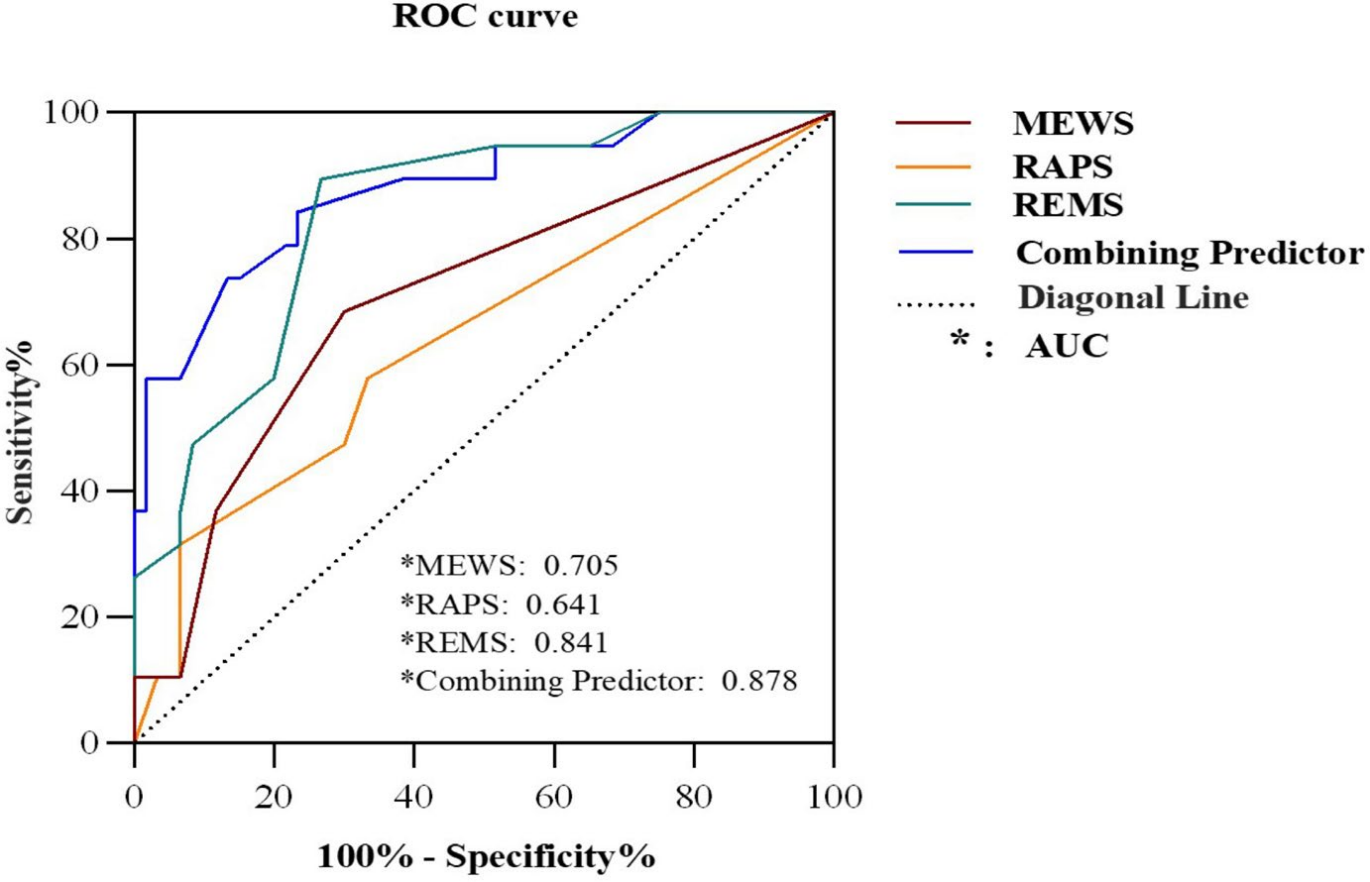
The receiver operating characteristic (ROC) curves for the Modified Early Warning Score (MEWS), Rapid Acute Physiology Score (RAPS), Rapid Emergency Medicine Score (REMS) and Combining predictors.

The binary logistic regression analysis demonstrated that MEWS, RAPS and REMS had a statistically significant ability to predict mortality in COVID‐19 patients (*p* <.05; Table 3). The ROC curve analysis demonstrated that of the three scales, the REMS had the best ability to predict the prognosis of COVID‐19 patients, with an AUC of 0.841 (*p* <.001). The MEWS yielded an AUC of 0.705 (*p* =.007), and the RAPS yielded an AUC of 0.641 (*p* =.065). Combining Predictors yielded an AUC of 0.878 (*p* <.001) (Table 4, Figure 2). Comparisons of the AUC associated with the MEWS, RAPS, REMS and Combining Predictors yielded. Significant differences were between the MEWS and REMS (*p* =.043) and between the RAPS and REMS (*p* =.0008), but not between the MEWS and RAPS (*p* =.35) or between the Combining Predictors and REMS (*p* =.401; Table 5). Comparisons of the NRI associated with the MEWS, RAPS, REMS and Combining Predictors yielded. Compared with RAPS, the accuracy of MEWS was significantly improved by 37.1% (*p* =.03), and the IDI (0.092) > 0 (*p* =.046). The accuracy of REMS was improved by 22.7% compared with MEWS (*p* =.12), and the IDI(0.09) > 0 (*p* =.047). REMS was improved by 43.2% (*p* =.002) compared with RAPS, and the IDI(0.182) > 0 (*p* =.002). Compared with REMS, the accuracy of Combining Predictor was improved by 25% (*p* =.113) and the value of IDI(0.02) > 0 (*p* =.598) (Table 6).

**TABLE 3:** The binary logistic regression analysis of MEWS, RAPS, and REMS scores

Variable	B	*SE*	Wald	*p*	OR	OR 95% CI	
						lower limit	upper limit
MEWS	0.732	0.37	3.916	**0.048**	2.079	1.007	4.291
RAPS	−1.002	0.401	6.263	**0.012**	0.367	0.167	0.805
REMS	0.768	0.227	11.444	**0.001**	2.156	1.382	3.365

**TABLE 4:** The AUCs and statistical parameters of the three scoring scales and combining predictors

Parameter	MEWS	RAPS	REMS	Combining predictors
AUC	0.705	0.641	0.841	0.878
*p*‐value	**0.007**	0.065	**<0.001**	**<0.001**
Cut‐off value	1.5	2.5	5.5	6.27
Youden index (max)	0.384	0.249	0.627	0.609
Sensitivity	0.68	0.32	0.9	0.84
Specificity	0.7	0.933	0.73	0.77
+LLR	2.28	4.72	3.35	3.61
−LLR	0.45	0.73	0.14	0.21

Note

AUC, area under the receiver operating characteristic curve; MEWS, Modified Early Warning Score; RAPS, Rapid Acute Physiology Score; REMS, Rapid Emergency Medicine Score.

**TABLE 5:** Comparisons between the AUCs of the MEWS, RAPS, REMS and combining predictors

*Z* Test	Difference between areas	*SE*	95% CI	*Z* statistic	*p*
RAPS vs. MEWS	0.0636	0.068	−0.0698 to 0.197	0.934	0.350
MEWS vs. REMS	0.136	0.067	0.00381 to 0.268	2.016	**0.043**
RAPS vs. REMS	0.2	0.059	0.0825 to 0.317	3.342	**0.0008**
REMS vs. combining predictor	0.0545	0.056	−0.0734 to 0.187	0.965	0.401

Note

AUC, area under the receiver operating characteristic curve; MEWS, Modified Early Warning Score; RAPS, Rapid Acute Physiology Score; REMS, Rapid Emergency Medicine Score; SE, standard error; CI, confidence interval.

**TABLE 6:** Comparisons between the NRI and IDI of the MEWS, RAPS, REMS and combining predictors

		Value	95% CI Lower	95% CI Upper	*p*
RAPS vs. MEWS	NRI	0.371	−0.178	0.604	**0.03**
	IDI	0.092	−0.011	0.164	**0.046**
MEWS vs. REMS	NRI	0.227	−0.114	0.632	0.12
	IDI	0.09	−0.078	0.286	**0.047**
RAPS vs. REMS	NRI	0.432	0.043	0.713	**0.002**
	IDI	0.182	0.024	0.347	**0.002**
REMS vs. combining predictor	NRI	0.25	−0.082	0.575	0.113
	IDI	0.02	−0.094	0.119	0.598

Note

NRI, Net Reclassification Improvement; IDI, Integrated Discrimination Improvement; MEWS, Modified Early Warning Score; RAPS, Rapid Acute Physiology Score; REMS, Rapid Emergency Medicine Score; CI, confidence interval

## 4 DISCUSSION

COVID‐19, a novel infectious disease caused by SARS‐CoV‐2 (Andersen et al., 2020), was first identified in December 2019 in Wuhan, the capital of Hubei Province in China. Since then, the disease has spread globally, resulting in the ongoing 2019–20 COVID‐19 pandemic (Hui et al., 2020). The common symptoms of COVID‐19 include fever, cough and dyspnoea, while some patients may also present with muscle pain, sputum production, diarrhoea, pharyngitis, anosmia and abdominal pain. Although the majority of cases result in mild symptoms, some progress to viral pneumonia and multi‐organ failure that particularly affects the lung (Chen et al., 2020; Wan et al., 2020). Currently, COVID‐19 is diagnosed mainly by viral nucleic acid detection tests and lung computed tomography scans. Although clinicians also use parameters such as the respiratory frequency, heart rate, body temperature, arterial oxygen partial pressure and indexes such as the white blood cell count, CRP concentration and APACHE II score to classify COVID‐19 cases according to severity, these prognostic factors are not specific. Moreover, the APACHE II scale is complex and requires a long time; therefore, it is not suitable for the rapid prognostic evaluation of a COVID‐19 patient. The identification of prognostic factors that could indicate the severity of COVID‐19 at an early stage would both improve patients’ outcomes and reduce the mortality rate. As no existing scale is available specifically to determine the severity of COVID‐19 at an early stage, we compared the abilities of three emergency rapid scoring scales and combined predictors to evaluate the prognosis of COVID‐19 patients. Our results identified the REMS as the best performing scale in this population.

SARS‐CoV‐2 infects host cells through interactions between the viral surface spike S protein and angiotensin‐converting enzyme 2 (ACE2) on the surfaces of host cell membranes. ACE2 is widely expressed in various tissues of human body, especially the alveolar epithelium, small intestinal epithelium and vascular endothelial cells. Upon entering the host cell, the virus replicates, and is released and simultaneously stimulates the host immune defence responses (Zhang, Penninger, et al., 2020). Studies have observed normal or decreased white blood cell counts, decreased lymphocyte counts and increased serum C‐reactive protein, creatinine and uric acid concentrations in some patients with early‐stage COVID‐19 (Huang et al., 2020.). In the present study, the patient's age, respiratory frequency, systolic blood pressure, oxygen saturation level, and white blood cell and lymphocyte counts were found to differ significantly between subjects in the surviving and deceased groups. According to previous studies, underlying medical conditions such as hypertension, lung disease, diabetes, cancer and immunocompromised status were important factors affecting the prognosis of COVID‐19. Similarly, we found that in addition to age, respiratory frequency, lymphocyte count and blood oxygen saturation level, the presence of a chronic underlying disease was an independent risk factor for mortality in COVID‐19 patients.

Of the scales evaluated in this study, the MEWS is an internationally recognized and effective early warning scoring method used to determine the severity of a disease condition. The MEWS includes evaluations of the patient's body temperature, heart rate, systolic blood pressure, respiratory rate and consciousness. However, our study indicated that the MEWS yielded a sensitivity and specificity of only 68% and 70%, respectively, for the prognostic evaluation of COVID‐19. The RAPS can be used to assess the mortality risk of patients in the ICU or those with severe trauma during transportation. This scale includes four parameters: blood pressure, pulse, respiratory frequency and Glasgow coma scale (GCS) score. However, the cut‐off value, sensitivity and specificity of the RAPS differ among disease types (Hung et al., 2017; Imhoff et al., 2014; Olsson, 2004). In our research, the RAPS yielded a very high specificity of 0.933 but a sensitivity of only 0.32.

The REMS was first applied by Olsson and Lind in 2003 to predict the mortality of patients with severe disease in an emergency internal medicine department (Olsson et al., 2004). This scoring system is ideal for rapid prognostication in an emergency department, and it features the advantages of a simple collection of observed indicators, the provision of relatively reliable information and the ability to enable an emergency department to determine a patient's prognosis at an early stage (Olsson et al., 2004). The REMS evaluate six indicators: heart rate, systolic blood pressure, respiratory frequency, GCS score, age and oxygen saturation level. In our study, the REMS yielded a sensitivity and specificity of 0.9 and 0.73, respectively, as well as a significantly greater AUC than those yielded by the RAPS and MEWS. +LLR and − LLR combined the advantages of sensitivity, specificity, positive predictive value and negative predictive value, and was not affected by the prevalence rate, so it was a relatively stable comprehensive index. The greater the +LLR, the greater the probability of true positive when the test result is positive. The smaller the −LLR, the more likely the test result was true negative. For RAPS, MEWS, REMS and Combining Predictor, the positive predictive value of MEWS was the largest and the negative predictive value of REMS was the smallest. If MEWS was used as the prediction model, although the accuracy of true positive was the highest, the accuracy of true negative was the smallest at the same time; The REMS model was not as accurate as MEWS in judging true positives, but it had the highest accuracy in predicting true negatives. While Combining Predictor was at a relatively balanced level. These findings suggest that the REMS can be used as a rapid assessment tool for the prognostication of patients with COVID‐19 in an emergency setting. We further determined that an old age and low oxygen saturation level are risk factors for mortality in COVID‐19 patients. As the REMS evaluation index includes both items, we speculate that therefore the REMS demonstrated a better prognostic ability in our study. We additionally combined the MEWS, RAPS and REMS and assessed the ability of this combination to predict the prognosis of patients. This combination yielded an AUC of 0.878, as well as a sensitivity and specificity of 0.84 and 0.77, respectively. Although this represents a slight improvement over the results obtained with the REMS alone, the difference was not significant.

Although AUC has been widely used in the evaluation of disease prediction models, AUC reveals the situation when all points on the ROC curve are used as cut‐off values. In real clinical practice, we usually only select an appropriate cut‐off point. At the same time, when we compare the prediction abilities of two models, especially when we want to compare whether the prediction ability of the models is improved after introducing new indicators into the models, it is sometimes difficult to significantly improve AUC with the new indicators. The increment of AUC is not obvious, and its significance is not easy to understand. In this case, we need to use NRI to compare the predictive ability of different models (Pencina et al., 2011) (Leening et al., 2014). NRI evaluates the probability that when the two models adopt the optimal cut‐off point for prediction, compared with the old model, the new model enables the individual prediction result to be improved. Pencina et al. (2008) obtained a new prediction model after adding HDL to the classical model and evaluated the model's ability to predict the risk of coronary heart disease in the next 10 years. The researchers first compared the ROC curves of the new and old models, and the results showed that the AUC was 0.774 and 0.762, respectively. The AUC of the new prediction model increased by 0.012 after adding HDL‐C, and the difference was not statistically significant (*p* =.092). However, they subsequently calculated the NRI value, and the results showed that the two models had significant statistical differences for the accuracy of individual prediction results. It indicated that the prediction ability of the new model was improved compared with the old model, and the proportion of correct classification was increased by 12.1%. Therefore, when the difference in AUC is not significant, the NRI can determine whether the new model is more advantageous. In our study, there was no significant difference in AUC between RAPS and MEWS, but NRI suggested that MEWS had better predictive power for individual outcomes. Therefore, NRI is more sensitive and easier to understand than AUC. However, NRI cannot examine the overall improvement of the model. In this case, we can choose another indicator: IDI. IDI reflects the change in the predicted probability difference between the two models and is calculated based on the predicted probability of the disease model for individual (Kerr et al., 2011). The larger the IDI is, the better the prediction ability of the new model will be indicated (Pencina et al., 2012). Like NRI, if IDI>0, it is positive improvement, indicating that the prediction ability of the new model is improved compared with that of the old model. If IDI<0, it is negative improvement, and the prediction ability of the new model is decreased. If IDI=0, it is considered that the new model is not improved. Compared with RAPS and MEWS, the IDI value of REMS model was greater than 0 and showed statistical difference. There were no differences when compared with the Combining Predictor. Therefore, the REMS model is the best choice among all models.

In summary, when comparing the two prediction models, in addition to the traditional AUC, NRI and IDI can also be given at the same time, to show the improvement of the new model in a more comprehensive and three‐dimensional manner, like the multi‐point perspective in painting techniques. When the conclusions of the three methods are basically consistent, it is proved that the reliability and accuracy of the model are better. In this study, the results of AUC, NRI and IDI were basically consistent, thus proving the stability and reliability of our model.

## 5 CONCLUSION

We determined that the REMS exhibited a better prognostic ability than the MEWS and RAPS when applied to patients with COVID‐19. Moreover, an old age, low blood oxygen saturation level and decreased lymphocyte count, which are included in the REMS, were identified as independent risk factors for mortality in COVID‐19 patients. We conclude that the REMS can be used as a rapid scoring tool for the early assessment of disease severity in COVID‐19 patients.

## 6 STUDY LIMITATION

This study was limited by a small sample size and a lack of details regarding the classification of chronic diseases, which made it difficult to determine which chronic diseases truly affect the prognosis of COVID‐19 patients. Moreover, our analysis of the MEWS, RAPS and REMS revealed that not all of the included items were identified as independent risk factors affecting prognosis, and consequently none of the scales yielded sensitivity and specificity values higher than 90%. Future studies should aim to expand the sample size, identify additional effective indicators and develop a new scale specifically for the evaluation of COVID‐19 patients.

## CONFLICT OF INTEREST

The authors declare no conflict of interest.

## AUTHOR CONTRIBUTIONS

Hai Hu: collection of data, writing up of article; Weili Kong: analysis of data, writing up of article, revising the article; Ni Yao and Yanru Qiu: collection of data; Rong Yao: statistics and analysis of data and answer reviewers’ questions. All authors read and approved the final manuscript.
