# Supplementary material for: Prognostic value of three rapid scoring scales and combined predictors for the assessment of patients with coronavirus disease 2019
Source: Nurs Open. 2021 Jun 3;9(3):1865–72. doi: 10.1002/nop2.934 (PMC8242648; doi:10.1002/nop2.934)
Supplement: Supplementary file 1 [file NOP2-9-1865-s001.docx]

**Supplementary material:**

**Table1 RAPS Scale**

|  | Score |  |  |  |  |
| --- | --- | --- | --- | --- | --- |
| Variable | 0 | +1 | +2 | +3 | +4 |
| PR（/min) | 70-109 |  | 55-69 | 40-54 | ≤39 |
|  |  |  | 110-139 | 140-179 | ≥180 |
| MAP(mmHg) | 70-109 |  | 50-69 | 130-159 | ≤49 |
|  |  |  | 110-129 |  | ≥160 |
| RR(/min） | 12-24 | 10-11 | 6-9 |  | ≤5 |
|  |  | 25-34 |  |  | ≥50 |
| GCS | ≥14 | 11-13 | 8-10 | 5-7 | ≤4 |

PR,pulse rate;MAP,mean arterial pressure;RR,repiratory rate;GCS,Glasgow Coma Scale.

**Table2 REMS Scale**

|  | **Score** | | | | | | |
| --- | --- | --- | --- | --- | --- | --- | --- |
| **Variable** | 0 | +1 | +2 | +3 | +4 | +5 | +6 |
| **Age(years)** | <45 |  | 45-54 | 55-64 |  | 65-74 | >74 |
| **PR(mmHg)** | 70-109 |  | 55-69 | 40-54 | ≦39 |  |  |
|  |  |  | 110-139 | 140-179 | >179 |  |  |
| **MAP(mmHg)** | 70-109 |  | 50-69 | 130-159 | ≦49 |  |  |
|  |  |  | 110-129 |  | >159 |  |  |
| **RR(/min)** | 12-24 | 10-11 | 6-9 | 35-49 | <5 |  |  |
|  |  | 25-34 |  |  | >49 |  |  |
| **GCS** | 14 or 15 | 11-13 | 8-10 | 5-7 | 3 or 4 |  |  |
| **SpO_2_** (%) | >89% | 86-89% |  | 75-85 | <75 |  |  |

PR, pulse rate; MAP, mean arterial pressure; RR, respiratory rate; GCS, Glasgow Coma Scale; SpO_2_, peripheral oxygen saturation.

**Table3 MEWS Scale**

|  | **Score** | | | |
| --- | --- | --- | --- | --- |
| **Variable** | 0 | +1 | +2 | +3 |
| **Systolic BP (mmHg)** | 101-199 | 81-100 | 71-80 | <70 |
|  |  |  | ≦200 |  |
| **Heart rate (/min)** | 51-100 | 41-50 | ≦40 | ≧130 |
|  |  | 101-110 | 111-129 |  |
| **Respiratory rate (/min)** | 9-14 | 15-20 | 21-29 | ≧30 |
|  |  |  | <35 |  |
| **Temperature (℃)** | 35-38.4 |  | ≧38.5 |  |
| **AVPU score** | Alert | Reacts to | Reacts to | Unresponsive |
|  |  | Voice | Pain |  |
